# Urbanization favors the proliferation of *Aedesaegypti* and *Culexquinquefasciatus* in urban areas of Miami-Dade County, Florida

**DOI:** 10.1038/s41598-021-02061-0

**Published:** 2021-11-26

**Authors:** André B. B. Wilke, Chalmers Vasquez, Augusto Carvajal, Maday Moreno, Douglas O. Fuller, Gabriel Cardenas, William D. Petrie, John C. Beier

**Affiliations:** 1 Department of Public Health Sciences, Miller School of Medicine, University of Miami, 1120 Northwest 14th Street, Miami, FL 33136 USA; 2 Miami-Dade County Mosquito Control Division, Miami, FL USA; 3 Department of Geography and Regional Studies, University of Miami, Coral Gables, FL USA

**Keywords:** Ecological epidemiology, Ecosystem ecology, Evolutionary ecology

## Abstract

Urbanization processes are increasing globally. Anthropogenic alterations in the environment have profound effects on biodiversity. Decreased biodiversity due to biotic homogenization processes as a consequence of urbanization often result in increased levels of mosquito vector species and vector-borne pathogen transmission. Understanding how anthropogenic alterations in the environment will affect the abundance, richness, and composition of vector mosquito species is crucial for the implementation of effective and targeted mosquito control strategies. We hypothesized that anthropogenic alterations in the environment are responsible for increasing the abundance of mosquito species that are adapted to urban environments such as *Aedes* *aegypti* and *Culex* *quinquefasciatus*. Therefore, our objective was to survey mosquito relative abundance, richness, and community composition in Miami-Dade County, Florida, in areas with different levels of urbanization. We selected 24 areas, 16 remote areas comprised of natural and rural areas, and 8 urban areas comprised of residential and touristic areas in Miami-Dade County, Florida. Mosquitoes were collected weekly in each area for 24 h for 5 consecutive weeks from August to October 2020 using BG-Sentinel traps baited with dry ice. A total of 36,645 mosquitoes were collected, from which 34,048 were collected in the remote areas and 2,597 in the urban areas. Our results show a clear and well-defined pattern of abundance, richness, and community composition according to anthropogenic modifications in land use and land cover. The more urbanized a given area the fewer species were found and those were primary vectors of arboviruses, *Ae.* *aegypti* and *Cx.* *quinquefasciatus*.

## Introduction

There are over one billion cases of mosquito-borne diseases reported worldwide every year^1^*.* The epidemiology of mosquito-borne diseases is affected by how modifications of the natural environment alter the interactions between vector mosquito species, hosts, and pathogens^2^. Anthropogenic changes in the environment in the form of climate change, urbanization, and biodiversity loss favor the proliferation of some vector mosquito species, such as *Aedes* *aegypti* and *Aedes* *albopictus*, and are important drivers for arbovirus transmission in urban areas^3–11^.

The epidemiology of mosquito-borne diseases continues to be uncertain since the extent to which mosquito species are dispersing across spatiotemporal scales and how urbanization processes are affecting their presence and abundance remains unclear^2,12,13^*.* Recent estimates have shown that 129 of the world’s countries and territories have conducive environments for the proliferation of mosquito vector species and are at risk of dengue transmission^1,14^*.*

Urbanization processes are ubiquitous in the contemporary world. Land use and land cover transformation to better suit the needs of the human population have led to the degradation of natural environments^15,16^. The human footprint can be found in most areas of the planet, including uninhabited areas^17–19^. The human population continues to grow and is expected to reach 8.5 billion people in 2030, 10 billion in 2050, and 11 billion in 2100^20^.

The urbanization of natural areas unavoidably leads to habitat fragmentation and shifts in resource availability^15^. The availability of resources in urban areas, and most importantly, the absence of resources that were available prior to their urbanization are responsible for either supporting or halting the proliferation of populations of vector mosquito species^3,21–23^. Many vector mosquito species are abundant in natural areas but due to their ecology and behavior are unable to invade and colonize urban areas^9,24^. On the other hand, mosquito vectors of anthroponotic vector-borne diseases represent a more pressing matter from the epidemiological standpoint due to their increased contact with humans^25,26^. Therefore, understanding how mosquito community composition and abundance are impacted by urbanization is key for the development of effective mosquito control strategies.

Many drivers are responsible for the proliferation of mosquito vectors of anthroponotic vector-borne diseases in urban areas. A previous study showed that, in Africa, urbanization processes are increasing the preference of *Ae.* *aegypti* to humans, being mainly driven by two factors: dry season intensity and human population density^27^. Furthermore, the levels of human exposure to mosquito vector species, especially in populations that spend a disproportioned amount of time outdoors such as the construction workforce^28^, was found to be an important driver for Zika virus transmission^29,30^. In this context, many socio-ecological drivers are important for mosquito proliferation in urban areas and play an important role in disease transmission^31,32^. Low-income neighborhoods and underserved populations are often more vulnerable to bites of mosquito vectors and consequently are at a higher risk of being exposed to arboviruses^33–35^.

Understanding the processes by which mosquito vector species adapt and thrive in urban environments is vital not only for the implementation of mosquito control strategies but also for improving and guiding policy to prevent outbreaks^22,36,37^*.* This is especially true for arboviral diseases such as dengue, Zika, and chikungunya, which are transmitted primarily by mosquito vector species that can thrive in urban environments such as *Ae.* *aegypti* and *Ae.* *albopictus*^38–40^*.*

Miami-Dade County, Florida has been the most affected county in the contiguous United States by *Aedes*-borne diseases^41–46^. Multiple introductions of the Zika virus to Miami-Dade in 2016^47^, have led to 256 locally transmitted human cases^48^, and in 2020, 6 cases of locally transmitted dengue virus were reported in Miami-Dade by the Florida Department of Health^49^. Furthermore, the Florida Department of Health reported 59 human cases of West Nile virus in Miami-Dade in 2020^50^, most of them reported in highly urbanized areas inhabited by underserved populations. Miami-Dade has suitable conditions for the proliferation of mosquitoes^3,9^. It has a warm climate and abundant rainfall added to rapid urban development to accommodate growing immigrant populations as well as transient underserved populations^51^.

Vector mosquito species such as *Ae.* *aegypti* and *Cx.* *quinquefasciatus* are abundant in Miami-Dade year-round^9^, and are important mosquito vectors of arboviruses in urban areas^52–54^. These species are able to exploit and benefit from a vast range of widely available resources that are present in urban areas^3,22,55^. Consistent with global population trends, current predictions indicate that approximately 700,000 people are expected to move to Miami-Dade by 2030^56^. Accordingly, Miami-Dade is experiencing increased levels of urbanization, and many natural areas are being transformed into urban areas^57^.

Understanding how such anthropogenic alterations in the environment will affect the abundance, richness, and composition of vector mosquito species is crucial for the implementation of effective and targeted mosquito control strategies. We hypothesized that urbanization processes are responsible for increasing the abundance of mosquito vectors of anthroponotic vector-borne diseases (e.g., Zika, chikungunya, dengue) by favoring mosquito species that are adapted to urban environments such as *Ae.* *aegypti* and *Cx.* *quinquefasciatus*. Therefore, our objective was to assess differences in the mosquito community composition between urbanized and natural and rural areas in Miami-Dade County, Florida.

## Results

Mosquitoes were collected in 24 collection sites across Miami-Dade County, from which 16 were located in remote areas and 8 in urban areas. A total of 36,645 mosquitoes were collected, from which 34,048 were collected in the remote areas and 2,597 in the urban areas. A total of 26 species were collected in the remote sites and 20 in the urban sites. The species richness per collection site ranged from 8 to 19 species among the remote sites with an average of 11 species per site, whereas among the urban sites the species richness ranged from 3 to 18 with an average of 6 species per site. However, the urban site U8 is a new urban development and borders natural areas. If the urban site U8 had been removed from the analyses the species richness among the urban sites would have ranged from 3 to 7 species with an average of 5 species per site.

All species collected in the urban sites were also collected in the remote sites. However, the opposite was not true, *Aedes* *bahamensis*, *Anopheles* *atropos*, *Coquillettidia* *perturbans*, *Psorophora* *ferox*, *Uranotaenia* *lowii*, and *Uranotaenia* *sapphirina* were collected in the remote sites but not on the urban sites. The most abundant species in the remote areas were *Cx.* *nigripalpus* (13,338 females and 3 males), followed by *Anopheles* *crucians* (6,509 females and 7 males) and *Culex* *erraticus* (6,086 females and 18 males). On the other hand, the most abundant species in the urban areas were *Ae.* *aegypti* (762 females and 399 males), followed by *Cx.* *nigripalpus* (547 females and 7 males) and *Cx.* *quinquefasciatus* (247 females and 185 males) (Table 1).

**Table 1:** Total number of mosquitoes collected at the remote and urban collection sites in Miami-Dade County, Florida. *F* female, *M* male.

Collection Site	*Ae.* *aegypti*		*Ae.* *albopictus*		*Ae.* *atlanticus*	*Ae.* *bahamensis*	*Ae.* *infirmatus*	*Ae.* *taeniorhynchus*		*Ae.* *tortilis*	*Ae.* *triseriatus*	*An.* *atropos*	*An.* *crucians*		*An.* *quadrimaculatus*		*Cq.* *perturbans*	*Cx.* *coronator*	*Cx.* *erraticus*		*Cx.* *interrogator*	*Cx.* *nigripalpus*		*Cx.* *quinquefasciatus*		*De.* *cancer*	*Ma.* *dyari*	*Ma.* *titillans*	*Ps.* *columbiae*	*Ps.* *ferox*	*Ur.* *lowii*	*Ur.* *sapphirina*	*Wy.* *mitchellii*	*Wy.* *vanduzeei*	Species Richness	Relative Abundance
	F	M	F	M	F	F	F	F	M	F	F	F	F	M	F	M	F	F	F	M	F	F	M	F	M	F	F	F	F	F	F	F	F	F		
R1	8	1		1			2	1					122				2	21	70	3		119		6						1	2				12	359
R2	2						1	6					156		47		2		328	5		52		11			6	7	4						12	627
R3	4	2	16	3	328		5	12		10	2		103				1		56	1		567		3	2				53	1					14	1,169
R4		41			4			16					90		500	1			363			946	1						1						8	1,963
R5	29	51	12	2	3			29					16	5	21				71			735		15	39				13						10	1,041
R6	2	18	1					33					103	2	61	5						800		19	38				5						8	1,087
R7					3			277				12	915		4				79			1,814							2		1	1			10	3,108
R8		4			2,484			69		2			15		17				2,788			7,092					694	29	6				80	160	13	13,440
R9								137					682		109		3		222			164					13	2						4	9	1,336
R10								68					1,078				1	1	472	6		24						1			4				8	1,655
R11		4			1			56					716		15				1,070	3		18				3			2						9	1,888
R12								115					2,140				1	1	208			17	1		3						2				8	2,488
R13		1	14	7		14		35		24		1	20		17			14	20			85		9		2			8						14	271
R14			3			11		465		172	16		14		5			47	96		14	583		6	3	94	4	6	4	39			2	12	19	1,596
R15			2		2			98		67	4	21	60		170			2	11			142	1	8	2	62									13	652
R16	17	18	8	1				17		549			279		13			19	232			180		5	5	15		10							12	1,368
**Total**	**62**	**140**	**56**	**14**	**2,825**	**25**	**8**	**1,434**		**824**	**22**	**34**	**6,509**	**7**	**979**	**6**	**10**	**105**	**6,086**	**18**	**14**	**13,338**	**3**	**82**	**92**	**176**	**717**	**55**	**98**	**41**	**9**	**1**	**82**	**176**	**26**	**34,048**
U1	16	14																				7		37	11									9	4	94
U2	154	159	1															1				1		73	65										5	454
U3	139	108																				1		40	45										3	333
U4	169	27						2		1												1		10	4	1									6	215
U5	7																					2		8											3	17
U6	88	14						2			14											3		13	24								1	46	7	205
U7	151	57						48		29									8			2		15	15									15	7	340
U8	38	20	4		99		1	36	3	14			11		13			15	38		2	530	7	51	21	1	8	25	1					1	18	939
**Total**	**762**	**399**	**5**		**99**		**1**	**88**	**3**	**44**	**14**		**11**		**13**			**16**	**46**		**2**	**547**	**7**	**247**	**185**	**2**	**8**	**25**	**1**				**1**	**71**	**20**	**2,597**
**Grand Total**	**824**	**539**	**61**	**14**	**2,924**	**25**	**9**	**1,522**	**3**	**868**	**36**	**34**	**6,520**	**7**	**992**	**6**	**10**	**121**	**6,132**	**18**	**16**	**13,885**	**10**	**329**	**277**	**178**	**725**	**80**	**99**	**41**	**9**	**1**	**83**	**247**	**26**	**36,645**

**Figure 1:**
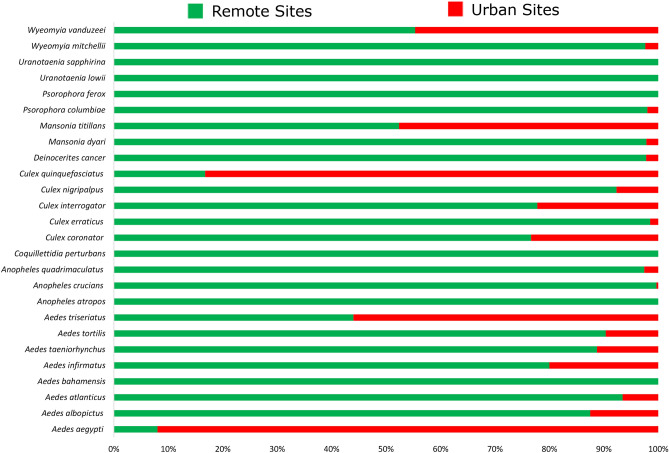
Relative proportion of mosquitoes collected at the remote and urban collection sites in Miami-Dade County, Florida.

The mean number of mosquitoes collected in the remote and urban collection sites revealed that most of the species were more commonly found in the remote areas. From the 26 species collected, only 5 species had a mean number higher than 20% in the urban areas, *Ae.* *aegypti*, *Ae.* *triseriatus*, *Cx.* *quinquefasciatus*, *Mansonia* *titillans*, and *Wyeomyia* *vanduzeei*. Furthermore, *Ae.* *aegypti* was the most commonly found species in urban areas with a mean value higher than 90%. On the other hand, *Ae.* *albopictus*, *An.* *crucians*, *Anopheles* *quadrimaculatus*, and *Cx.* *erraticus* were almost exclusively collected in the remote areas (Fig. 1).

**Figure 2:**
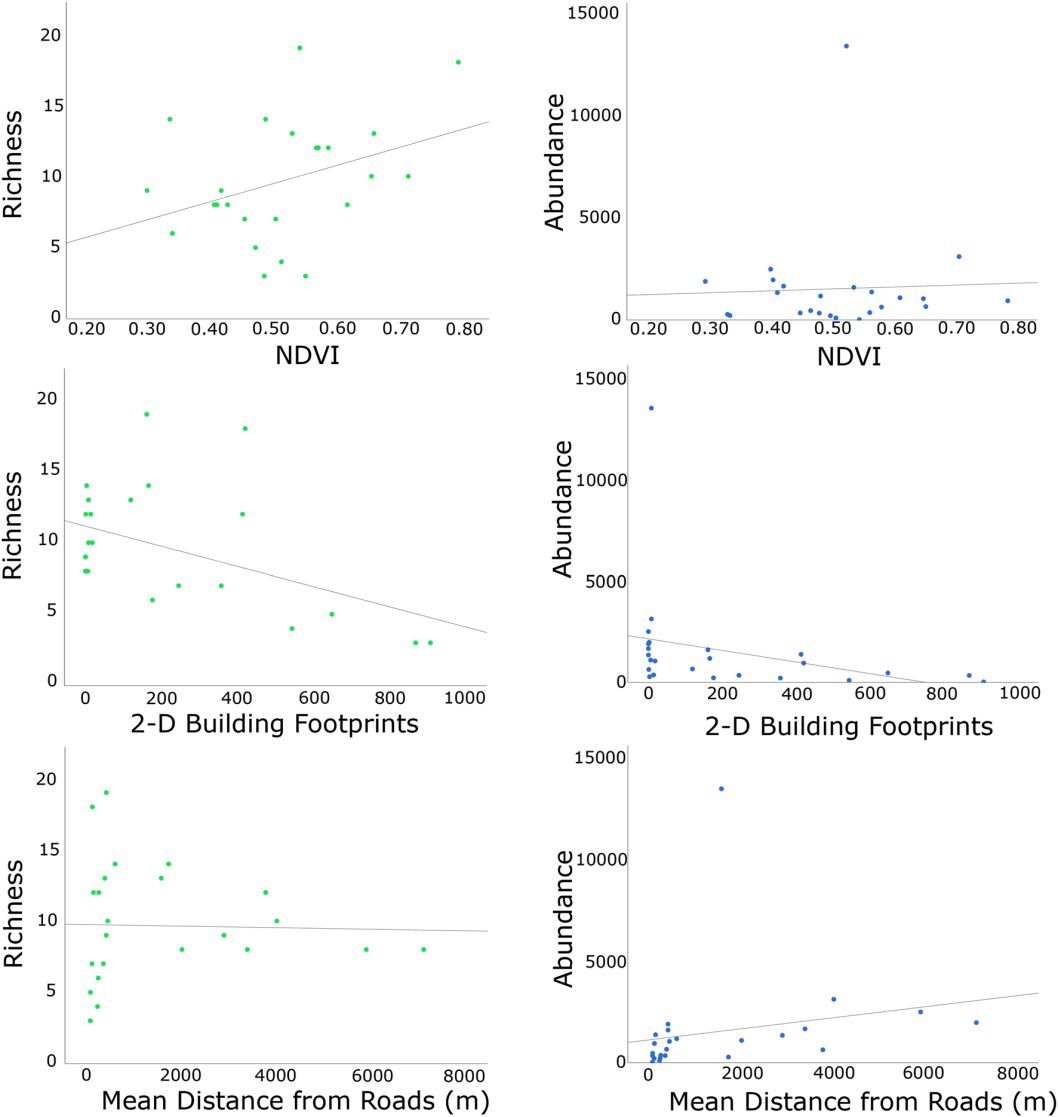
Impact of urbanization on species richness and mosquito relative abundance. Left: Species richness relationship with NDVI, building footprint, and mean distance from roads (in meters); Right: Relative abundance relationship with NDVI, building footprint, and mean distance from roads (in meters).

Our results indicate a robust pattern of mosquito relative abundance and species richness and composition between remote and urban collection sites. The remote collection sites had a higher species richness with many abundant species that were not commonly found in the urban collection sites such as *Aedes* *atlanticus*, *An.* *quadrimaculatus*, and *Cx.* *erraticus*. On the other hand, *Ae.* *aegypti* and *Cx.* *quinquefasciatus* were more commonly found in the urban collection sites. Furthermore, the species richness and abundance were higher in areas less impacted by urbanization, being inversely proportional to the presence of buildings and proximity to roads (Fig. 2).

**Figure 3:**
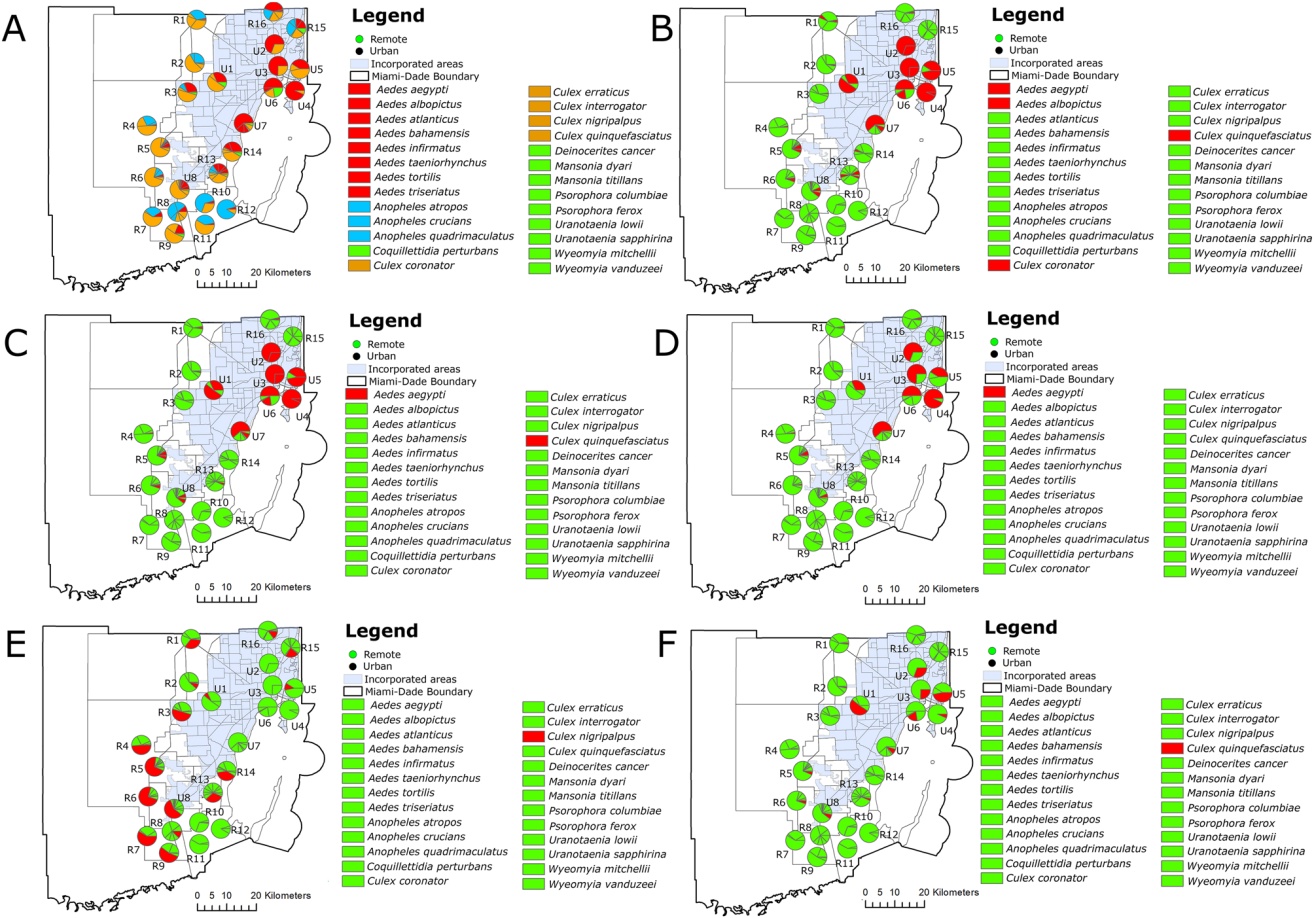
Percent composition of vector mosquito species in remote and urban collection site locations in Miami-Dade County, Florida. Species were organized by (**A**) red = *Aedes*, blue = *Anopheles*, yellow = *Culex*, green = *Coquillettidia*, *Deinocerites*, *Mansonia*, *Psorophora*, *Uranotaenia*, and *Wyeomyia*; (**B**) red = *Aedes* *aegypti*, *Aedes* *albopictus*, *Culex* *coronator*, and *Culex* *quinquefasciatus*, green = all other species; (**C**) red = *Aedes* *aegypti* *and* *Culex* *quinquefasciatus*, green = all other species; (**D**) red = *Aedes* *aegypti*, green = all other species; (**E**) red = *Culex* *nigripalpus*, green = all other species; and (**F**) red = *Culex* *quinquefasciatus*, green = all other species. The figure was produced using ArcGIS 10.2 (Esri, Redlands, CA), using freely available layers from the Miami-Dade County’s Open Data Hub—https://gis-mdc.opendata.arcgis.com/.

The analysis of the percent composition of vector mosquito species in the remote and urban collection sites revealed that the urban collection sites had fewer and more dominant mosquito vector species than the remote collection sites. *Aedes* *aegypti* and *Cx.* *quinquefasciatus* comprised most of the mosquitoes collected in the urban sites with *Ae.* *aegypti* being the most abundant species collected in these sites except collection site U8, which was located in a natural-urban transition zone. On the other hand, *Ae.* *aegypti* and *Cx.* *quinquefasciatus* were not collected in 6 of the remote sites and apart from the remote sites R4 and R5 were collected in negligible numbers in the remote collection sites. On the remote collection sites, *Cx.* *nigripalpus* was the dominant species but not as dominant as *Ae.* *aegypti* and *Cx.* *quinquefasciatus* in the urban collection sites. These results showed a clear pattern of species richness and relative abundance in both the remote and urban collection sites according to the environment and respective resource availability. The mosquito community composition was substantially different in remote and urban collection sites disregarding geographical proximity as observed in the comparison between the collection sites U1 and R2 and R3, U2 and R15 and R16, and U7 and R14 (Fig. 3).

The Permutational Multivariate Analysis of Variance (PERMANOVA) yielded significant results for the comparison between mosquito species composition in remote and urban areas (F = 8.707; *P* = 0.0001). The PERMANOVA analysis also revealed significant differences in the mosquito species composition in areas with different land uses (F = 3.205; *P* < 0.0003). The subsequent SIMPER (Similarity Percentage) analysis comparing remote and urban areas showed that *Cx.* *nigripalpus*, *An.* *crucians*, *Cx.* *erraticus*, and *Ae.* *aegypti* contributed the most to the observed differences, whereas in the analysis considering different land uses *Cx.* *nigripalpus*, *An.* *crucians*, *Cx.* *erraticus*, and *Ae.* *atlanticus* contributed the most to the observed differences (Supplementary Table 2).

Results from the Moran's Index of Spatial Autocorrelation were not statistically significant failing to reject the null hypothesis, therefore, indicating that autocorrelation is not present in the samples analyzed here (Observed: − 0.002; Expected: − 0.043; Standard Deviation: 0.050; and *P*-value: 0.420). The Generalized Linear Mixed Methods (GLMM) regression results showed a statistically significant association between the Normalized Difference Vegetation Index (NDVI) and the mean distance from major roads and their interaction with species richness (Table 2).

**Table 2:** Generalized linear mixed methods (GLMM) regression results.

Covariates	Species Richness			Relative Abundance		
	Wald Chi-Square	df	*P* value	Wald Chi-Square	df	*P* value
Model	0.277	1	0.598	7.603	1	**0.01**
NDVI	17.78	1	** < .001**	2.734	1	0.1
Building Footprint	0.527	1	0.468	0.211	1	0.65
Mean Distance from Roads	12.422	1	** < .001**	0.758	1	0.38
NDVI * Building Footprint	0.173	1	0.677	1.637	1	0.2
NDVI * Mean Distance from Roads	15.639	1	** < .001**	0.65	1	0.42
Building Footprint * Mean Distance from Roads	3.158	1	0.076	1.756	1	0.19

Species richness and relative abundance served as the dependent variables and were analyzed using a normal distribution for each urbanization measurement (Normalized Difference Vegetation Index (NDVI), 2-D Building Footprint, and the mean distance from major roads) and their interaction as independent variables, and week as a random effect with collection sites nested in time (weeks).

## Discussion

Our results show a clear and well-defined pattern of abundance, richness, and community composition according to anthropogenic modifications in land use and land cover in Miami-Dade. The more urbanized a given area the fewer species were found and *Ae.* *aegypti* and *Cx.* *quinquefasciatus* were the most dominant species. Our study shows that land use and land cover transformation of natural areas into urban areas of South Florida can affect mosquito relative abundance, richness, and community composition by favoring mosquito vector species that are adapted to thrive in urban environments. As a consequence, human populations will have increased contact with mosquito vector species, especially *Ae.* *aegypti* and *Cx.* *quinquefasciatus,* increasing the risk of vector-borne disease transmission.

Our results also show that the community composition, species richness, and relative abundance were not related to the geographic proximity of the remote and urban collection sites. Even adjacent remote and urban collection sites had completely distinct mosquito community composition, species richness, and relative abundance. These results support the hypothesis that the availability of resources at the micro-geographic scale (i. e., neighborhood-level) is a major driver for the proliferation or decrease of populations of vector mosquito species according to their specific ecology and behavior^22,58^. For example, both *Ae.* *aegypti* and *Cx.* *quinquefasciatus* were collected in relatively high numbers in the remote areas R4 and R5. The remote area R4 is a conservation zone closed to the general public, however, an abandoned checkpoint flooded with rainwater was a potentially conducive habitat for the proliferation of *Ae.* *aegypti* and *Cx.* *quinquefasciatus*. A similar scenario was observed in the remote area R5, in which the presence of a human-made ditch to divert rainwater also made available suitable habitats for these vector species.

The transformation of natural environments into urban spaces and the increased density of urban land cover surfaces (e.g., asphalt, concrete, etc.) within existing urbanized locations is a major driver for biodiversity loss^6,59,60^. As a result, the steep increase in urbanization in the last decades has led to biodiversity loss on a global scale^15^.

In this context, the mosquito community composition is greatly affected by urbanization, in which mosquito abundance and species richness are significantly affected and tend to decrease proportionally with urbanization levels^61–64^. However, some mosquito vector species can thrive in urban environments and greatly benefit from the resources made available by the rise of transient underserved populations, absence of natural predators, human-made aquatic habitats, increased human population density, and warmer temperatures due to global warming. *Aedes* *aegypti* and *Cx.* *quinquefasciatus* are among those species that can thrive in urban areas, and coincidentally are the primary vectors of chikungunya, dengue, yellow fever, West Nile, and Zika viruses^48,65–69^. This is in agreement with the results from the GLMM analysis, in which NDVI and mean distance from roads were significantly associated with reduced species richness.

Our results also revealed the presence of many mosquito vector species in the remote areas of Miami-Dade. *Anopheles* *quadrimaculatus*, the primary vector of human malaria in the southeast United States and *Cx.* *erraticus*, the bridge vector of Eastern Equine Encephalitis in the southern United States, were almost exclusively found in the remote areas and were only collected in high numbers in the urban area U8, a new urban development bordering natural areas. *Aedes* *albopictus* is not commonly found in urban areas in Miami-Dade County^9,22^, and is relegated to restricted and well-defined areas such as cemeteries^70^. Therefore, it was not unexpected that approximately 90% of *Ae.* *albopictus* specimens were collected in the remote areas. However, despite the epidemiological importance of these species, their distant relationship with humans and inability of invading and thriving in urban areas of Miami-Dade greatly decreases their relevance in disease transmission when compared with mosquito vectors of anthroponotic vector-borne diseases. In this context, as our results revealed, *Ae.* *aegypti* is considerably more abundant in urban areas and is the primary vector of arboviruses that use humans as amplification hosts without the need for bridge vectors and primary hosts, such as dengue and Zika, representing a much greater public health threat^71^.

Urbanization has a major impact on the epidemiology of vector-borne disease transmission. It not only provides all the resources necessary for the survival of vector mosquito species such as *Ae.* *aegypti* and *Cx.* *quinquefasciatus*, but also provides shelter from the elements. Urban features such as tire shops or underground subway stations may allow populations of vector mosquito species to survive scorching and freezing temperatures that normally would kill them^72,73^. Furthermore, these highly productive urban environments that are responsible for the proliferation of vector mosquitoes are often located in populous areas, increasing, even more, the contact between humans and mosquito vectors^28,70^.

The impact of urbanization in the proliferation of vector mosquito species and arbovirus transmission must be considered under the Integrated Vector Management (IVM) framework^74^. Environmental ordinances and good practices, as well as simple modifications in the urban built environment at the early stages of development, can substantially decrease human exposure to mosquito vectors by attenuating social inequities and consequently social determinants of health^75,76^.

Even though several new technologies for controlling vector mosquito species in urban areas are being developed (e.g., genetically modified mosquitoes and *Wolbachia*-infect mosquitoes), they have not been validated to be used on large scales under real-world conditions^77,78^, and further entomological and epidemiological validation is still needed before they can be included and implemented under the IVM framework^79^. In this context, the World Health Organization (WHO) recommendation to control vector mosquito populations relies on the removal of aquatic habitats for immature mosquitoes and targeted insecticide application when needed. However, the current levels of proliferation of vector mosquito species in urban areas mediated by an overabundance of resources^3,9,22^ make it virtually impossible to achieve the desired results of a safe mosquito abundance threshold to avoid arbovirus transmission^80^.

This study is not without limitations. We did not collect data across all weather and season variations that would have brought further insight into the natural variation in the mosquito community composition and abundance. We have collected mosquitoes using BG-Sentinel traps, which are the gold standard for collecting *Aedes* *Stegomyia* species. Even though BG-Sentinel traps have been proven effective to collect other less anthropophilic mosquito species and the fact that we have collected 26 mosquito species during this study, including many species that are notably not anthropophilic, we may have underestimated the presence and relative abundance of mosquito species that are less attracted by the BG-Sentinel traps.

## Conclusion

The relationships between mosquito vectors, human hosts, and pathogens are driven by environmental conditions. Vector-borne diseases are conditioned by the environment, and anthropogenic changes have a direct influence on their epidemiology^7^. Climate change and urbanization increase the risk of arbovirus transmission by increasing the presence and abundance of mosquito vector species, therefore, increasing their contact with human populations. The findings of this study shed light on the effect of urbanization on the community composition of mosquitoes by reducing species richness and increasing the abundance of *Ae.* *aegypti* and *Cx.* *quinquefasciatus* in a non-random process of biotic homogenization. Large urban areas hold diverse socio-ecological conditions that can be highly conducive to both mosquito vector proliferation and local arbovirus transmission. Miami-Dade is one of the most critical entry points into the United States, with an elevated influx of people arriving and departing from endemic areas. Miami-Dade is, therefore, a sentinel or harbinger of what other cities in the contiguous United States will experience this century with climate change, population growth, regional trade, and human movement. The findings from this study highlight the importance of understanding how anthropogenic changes in the environment create an overabundance of resources that are responsible for sustaining the invasion, spread, and colonization of urban areas by vector mosquito species.

## Methods

### Study design

**Figure 4:**
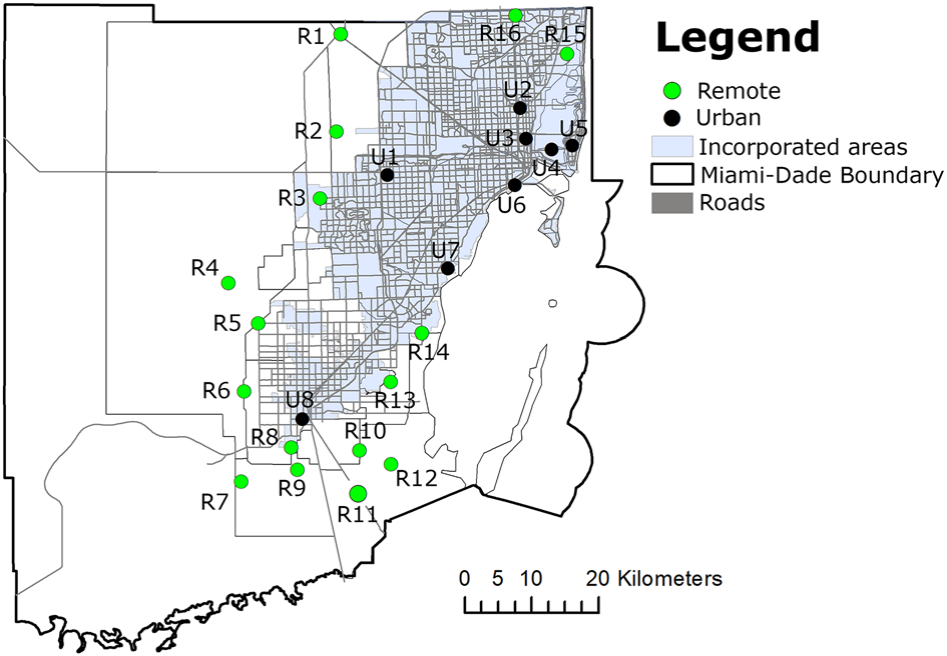
Map showing the location of the remote and urban collection sites in Miami-Dade, Florida (latitude, 25.761681; longitude, -80.191788). The figure was produced using ArcGIS 10.2 (Esri, Redlands, CA) using freely available layers from the Miami-Dade County’s Open Data Hub— https://gis-mdc.opendata.arcgis.com/.

In this study, we identified and selected 24 areas: (i) 16 remote areas with high normalized difference vegetation index (NDVI) values. These areas were comprised of natural and rural areas with low population density or complete absence of humans, and no or minimum urban development allowed; and (ii) 8 urban areas with low NDVI values comprised of a university campus, and residential, and touristic areas with high human population density in Miami-Dade County, Florida (Fig. 4). We quantified the change in the NDVI obtained from Landsat satellite imagery mapped at 30 m spatial resolution. NDVI, which ranges from − 1 to + 1, provides a direct measurement of photosynthetic activity and is positively correlated with moisture availability, evapotranspiration, and vegetation biomass^81^. NDVI values less than or equal to ~ 0.1 are typically associated with urbanized surfaces (e.g., pavement, bare soils, and rooftops) or water bodies. Thus, decreases in NDVI through time may indicate vegetation loss or degradation associated with urban and suburban development characteristics of South Florida. For example, Al Rifat and Liu^82^ found that urbanization in Miami-Dade County advanced at a rate of 96.57 km^2^ yr^*−*1^ between 1996–2001 but had decreased to 11.45 km^2^ yr^−1^ between 2011–2016 as available land areas for development became increasingly limited. Further, their analysis revealed that urbanized surfaces in Miami-Dade County also became more compact (i.e., dense) since the 1990s^82^.

We selected a cloud-free, atmospherically corrected Landsat from 2017 from https://earthexplorer.usgs.gov/ during the month of November, which coincides with the approximate start of the dry season in South Florida^82,83^. To assess vegetation conditions around the traps, we utilized GIS software to create 1-km buffers around each trap and calculated the mean and standard deviation (SD) of NDVI within 1-km radii, which coincides approximately with the maximum flight distance of many species collected at the traps. The NDVI for each trap buffer provides a measure of spatial variation in vegetation state around each trap. For traps that were proximate to the shore (i.e., within 1 km of a shoreline), we masked out any water portions of the radius so that only NDVI values from land areas were included in NDVI. The masking process reduced the sample area from 3.15 km^2^ for inland locations (i.e., greater than 1 km from shorelines) to a minimum of 0.76 km^2^ for one island site with an extensive shoreline (Table 3). Then, we used the same 1-km buffers around each trap to calculate the number of pixels with buildings in each buffer around the trap sites to create a 2-D Building Footprint as one measure of urban development. We also calculated the mean distance from major roads in meters for each of the buffers (i.e., mean for all the pixels that comprise a buffer) as a proxy for urbanization. The layers are freely available at the Miami-Dade County’s Open Data Hub— https://gis-mdc.opendata.arcgis.com/.

**Table 3:** Description of the remote and urban collection sites in Miami-Dade, Florida.

Collection Site	Location	Latitude	Longitude	Description	NDVI 2017	2-D Building Footprints	Mean Distance from Roads (m)
U1	Sweetwater	25.757452	− 80.376182	University	0.506	543	240
U2	Little River	25.844456	− 80.20358	Residential	0.465	648	90
U3	Wynwood	25.80472	− 80.196006	Touristic	0.479	868	89
U4	San Marino	25.791004	− 80.16252	Residential	0.334	176	254
U5	Convention Center	25.796003	− 80.135516	Touristic	0.544	907	86
U6	Vizcaya	25.744416	− 80.210485	Touristic	0.497	357	123
U7	Tarpon Dr	25.636065	− 80.297609	Residential	0.448	245	363
U8	Naranja	25.440493	− 80.48667	Residential	0.785	420	131
R1	Okeechobee	25.94113	− 80.43672	Natural	0.561	14	267
R2	West Doral	25.81421	− 80.44214	Natural	0.580	1	3,775
R3	Bird Road	25.72724	− 80.46365	Natural	0.481	166	611
R4	East Everglades	25.61732	− 80.58278	Natural	0.404	2	7,099
R5	Hainlin Mill	25.56462	− 80.54359	Rural	0.648	18	455
R6	Everglades Trail	25.47662	− 80.56225	Rural	0.610	6	2,016
R7	Southern Glades	25.35947	− 80.56582	Natural	0.706	8	4,012
R8	Alligator Farm	25.40366	− 80.50124	Rural	0.523	8	1,581
R9	Detention Center	25.37453	− 80.49284	Natural	0.411	0	2,898
R10	SW 137 Ave	25.40004	− 80.41216	Natural	0.421	0	3,390
R11	Card Sound	25.34158	− 80.41219	Natural	0.294	0	424
R12	Cooling Canals	25.38174	− 80.37111	Natural	0.400	0	5,892
R13	Air Force Base	25.48889	− 80.37162	Rural	0.330	3	1,733
R14	Black Point	25.55204	− 80.33118	Natural	0.535	161	424
R15	Oleta Park	25.915682	− 80.142643	Natural	0.652	119	393
R16	NW 207 St	25.96551	− 80.209737	Natural	0.564	413	154

### Collection of mosquitoes

Mosquitoes were collected from August to October 2020 using BG-Sentinel traps (Biogents AG, Regensburg, Germany) baited with dry ice^84^. Mosquitoes were collected weekly at each collection site for 24 h for 5 consecutive weeks. Traps were placed in shaded areas that were protected from direct solar radiation, wind, and precipitation to enhance mosquito collections. The collected mosquitoes were transported to the Miami-Dade County Mosquito Control Laboratory and subsequently morphologically identified to species using taxonomic keys^85^.

### Statistical analyses

To compare the mosquito species composition in remote and urban areas as well as in areas with different land uses we performed a PERMANOVA with 9,999 permutations based on Bray–Curtis distances^86,87^. First, we subsetted the data into two groups to compare the mosquito species composition between rural and urban areas, and then we subsequently subsetted the data into 5 groups according to land use: natural, rural, university, residential, and touristic areas. Then we used the SIMPER method for assessing which species has contributed the most to the observed differences between groups of samples^88^. Analyses were done using PAST v3.2^89^.

We assessed spatial autocorrelation between samples using Moran's Index of Spatial Autocorrelation using ArcMap v10.5, in which failing to accept the alternative hypothesis indicates autocorrelation is not present in the data^90^. We checked for multicollinearity between the covariates NDVI, 2-D Building Footprint, and the mean distance from major roads using the variance inflation factor (VIF) values. VIF values below 10.00 indicate the assumptions were met and the covariates were not colinear^91,92^. There was no collinearity between the covariates, as the highest VIF value yielded 1.433 (Supplementary Table 3). We used the Eta squared (η^2^) as the proportion of variance in the continuous target field explained by an effect to determine effect size^93^ (Supplementary Table 4). Then, we performed a Generalized Linear Mixed Methods (GLMM) regression using a normal distribution considering species richness and relative abundance as the dependent variables and each urbanization measurement (Normalized Difference Vegetation Index (NDVI), 2-D Building Footprint, and the mean distance from major roads) and their interaction as independent variables, and week as a random effect with collection sites nested in time (weeks). Statistical analyses were done in SPSS V28.0.

## Supplementary Information

Supplementary Information 1.

Supplementary Information 2.

Supplementary Information 3.

Supplementary Information 4.
