# Supplementary material for: Urbanization favors the proliferation of Aedesaegypti and Culexquinquefasciatus in urban areas of Miami-Dade County, Florida
Source: Sci Rep. 2021 Nov 26;11:22989. doi: 10.1038/s41598-021-02061-0 (PMC8626430; doi:10.1038/s41598-021-02061-0)
Supplement: Supplementary file 2 — Supplementary Information 2. [file 41598_2021_2061_MOESM2_ESM.docx]

Supplementary Table 2. SIMPER (Similarity Percentage) analysis of which species contributed the most to the observed differences comparing remote and urban areas and areas with different land uses.

| Remote vs Urban Areas |  |  |  |  |  |  | Areas with Different Land Uses |  |  |  |  |  |
| --- | --- | --- | --- | --- | --- | --- | --- | --- | --- | --- | --- | --- |
| Species | Av. dissim | Contrib. % | Cumulative % | Mean 1 | Mean 2 |  | Species | Av. dissim | Contrib. % | Cumulative % | Mean 1 | Mean 2 |
| *Culex nigripalpus* | 25.81 | 28.13 | 28.13 | 834 | 69.3 |  | *Culex nigripalpus* | 27.37 | 36.34 | 36.34 | 386 | 2.18E+03 |
| *Anopheles crucians* | 19.93 | 21.72 | 49.85 | 407 | 1.38 |  | *Anopheles crucians* | 14.86 | 19.73 | 56.07 | 530 | 40.3 |
| *Culex erraticus* | 13.33 | 14.53 | 64.38 | 382 | 5.75 |  | *Culex erraticus* | 12.41 | 16.48 | 72.55 | 269 | 720 |
| *Aedes aegypti* | 9.456 | 10.31 | 74.68 | 12.6 | 145 |  | *Aedes atlanticus* | 5.24 | 6.958 | 79.51 | 28.2 | 622 |
| *Aedes taeniorhynchus* | 4.648 | 5.067 | 79.75 | 89.6 | 11.4 |  | *Aedes taeniorhynchus* | 3.111 | 4.131 | 83.64 | 106 | 41.5 |
| *Anopheles quadrimaculatus* | 3.999 | 4.359 | 84.11 | 61.6 | 1.63 |  | *Anopheles quadrimaculatus* | 2.868 | 3.809 | 87.45 | 72 | 30.3 |
| *Aedes tortilis* | 3.543 | 3.862 | 87.97 | 51.5 | 5.5 |  | *Aedes tortilis* | 2.725 | 3.619 | 91.07 | 66.5 | 6.5 |
| *Culex quinquefasciatus* | 3.077 | 3.354 | 91.32 | 10.9 | 54 |  | *Mansonia dyari* | 1.238 | 1.645 | 92.71 | 1.92 | 174 |
| *Aedes atlanticus* | 3.058 | 3.333 | 94.66 | 177 | 12.4 |  | *Culex quinquefasciatus* | 1.143 | 1.518 | 94.23 | 4.5 | 30 |
| *Deinocerites cancer* | 0.8268 | 0.9011 | 95.56 | 11 | 0.25 |  | *Aedes aegypti* | 1.097 | 1.456 | 95.69 | 8.08 | 26.3 |
| *Wyeomyia vanduzeei* | 0.7675 | 0.8366 | 96.39 | 11 | 8.88 |  | *Deinocerites cancer* | 0.6316 | 0.8386 | 96.53 | 14.5 | 0.5 |
| *Culex coronator* | 0.6637 | 0.7234 | 97.12 | 6.56 | 2 |  | *Aedes albopictus* | 0.4782 | 0.6351 | 97.16 | 2.83 | 9 |
| *Aedes albopictus* | 0.4813 | 0.5246 | 97.64 | 4.38 | 0.625 |  | *Psorophora columbiae* | 0.4232 | 0.562 | 97.72 | 5.5 | 8 |
| *Psorophora columbiae* | 0.4664 | 0.5084 | 98.15 | 6.13 | 0.125 |  | *Culex coronator* | 0.4115 | 0.5465 | 98.27 | 7.58 | 3.5 |
| *Mansonia dyari* | 0.4513 | 0.4919 | 98.64 | 44.8 | 1 |  | *Wyeomyia vanduzeei* | 0.3102 | 0.4119 | 98.68 | 1.33 | 40 |
| *Mansonia titillans* | 0.2511 | 0.2737 | 98.92 | 3.44 | 3.13 |  | *Aedes bahamensis* | 0.2496 | 0.3314 | 99.01 | 0.917 | 3.5 |
| *Aedes bahamensis* | 0.21 | 0.2289 | 99.15 | 1.56 | 0 |  | *Wyeomyia mitchellii* | 0.1391 | 0.1848 | 99.2 | 0.167 | 20 |
| *Aedes triseriatus* | 0.205 | 0.2234 | 99.37 | 1.38 | 1.75 |  | *Anopheles atropos* | 0.134 | 0.178 | 99.38 | 2.75 | 0.25 |
| *Anopheles atropos* | 0.1773 | 0.1932 | 99.56 | 2.13 | 0 |  | *Mansonia titillans* | 0.133 | 0.1766 | 99.55 | 2.17 | 7.25 |
| *Psorophora ferox* | 0.1437 | 0.1567 | 99.72 | 2.56 | 0 |  | *Psorophora ferox* | 0.1199 | 0.1592 | 99.71 | 3.42 | 0 |
| *Culex interrogator* | 0.0565 | 0.06159 | 99.78 | 0.875 | 0.25 |  | *Aedes triseriatus* | 0.07138 | 0.09479 | 99.81 | 1.83 | 0 |
| *Coquillettidia perturbans* | 0.05602 | 0.06106 | 99.84 | 0.625 | 0 |  | *Coquillettidia perturbans* | 0.04043 | 0.05368 | 99.86 | 0.833 | 0 |
| *Aedes infirmatus* | 0.05198 | 0.05666 | 99.9 | 0.5 | 0.125 |  | *Culex interrogator* | 0.03949 | 0.05244 | 99.91 | 1.17 | 0 |
| *Wyeomyia mitchellii* | 0.05183 | 0.05649 | 99.95 | 5.13 | 0.125 |  | *Aedes infirmatus* | 0.03498 | 0.04645 | 99.96 | 0.667 | 0 |
| *Uranotaenia lowii* | 0.03986 | 0.04345 | 100 | 0.563 | 0 |  | *Uranotaenia lowii* | 0.02964 | 0.03936 | 100 | 0.75 | 0 |
| *Uranotaenia sapphirina* | 0.001831 | 0.001996 | 100 | 0.0625 | 0 |  | *Uranotaenia sapphirina* | 0.001741 | 0.002312 | 100 | 0.0833 | 0 |
