# Supplementary material for: Urbanization favors the proliferation of Aedesaegypti and Culexquinquefasciatus in urban areas of Miami-Dade County, Florida
Source: Sci Rep. 2021 Nov 26;11:22989. doi: 10.1038/s41598-021-02061-0 (PMC8626430; doi:10.1038/s41598-021-02061-0)
Supplement: Supplementary file 3 — Supplementary Information 3. [file 41598_2021_2061_MOESM3_ESM.docx]

**Supplementary Table 3. Variance inflation factor (VIF) values multicollinearity test.**

| Covariates | t | *P-*value | Tolerance | VIF |
| --- | --- | --- | --- | --- |
| (Constant) | 2.127 | **0.035** |  |  |
| NDVI | 6 | **0.001** | 0.977 | 1.024 |
| Building Footprint | -6.289 | **0.001** | 0.701 | 1.426 |
| Mean Distance from Roads | -2.803 | **0.006** | 0.698 | 1.433 |
