# Supplementary material for: Urbanization favors the proliferation of Aedesaegypti and Culexquinquefasciatus in urban areas of Miami-Dade County, Florida
Source: Sci Rep. 2021 Nov 26;11:22989. doi: 10.1038/s41598-021-02061-0 (PMC8626430; doi:10.1038/s41598-021-02061-0)
Supplement: Supplementary file 4 — Supplementary Information 4. [file 41598_2021_2061_MOESM4_ESM.docx]

**Supplementary Table 4.** **Effect size calculation based on the proportion of variance in the continuous target field explained by an effect.**

|  | NDVI | | Building Footprint | | Mean Distance from Roads | |
| --- | --- | --- | --- | --- | --- | --- |
|  | η | η^2^ | η | η^2^ | η | η^2^ |
| Richness | 0.69 | 0.476 | 0.685 | 0.469 | 0.674 | 0.455 |
| Abundance | 0.686 | 0.471 | 0.571 | 0.326 | 0.686 | 0.471 |
